# K-wires as a valid alternative to dual and single headless cannulated compression screw fixation of chauffeur fractures: a biomechanical comparison

**DOI:** 10.1007/s00068-025-02971-y

**Published:** 2025-10-28

**Authors:** Nicolas Catalin Ionut Ion, Bogdan-Axente Bocea, Ivan Zderic, Radu Sorin Fleaca, Mihai-Dan Roman, Cosmin-Ioan Mohor, R. Geoff Richards, Mark Lenz, Boyko Gueorguiev, Ludmil Drenchev, Tatjana Pastor

**Affiliations:** 1 AO Research Institute Davos, Davos, Switzerland; 2 Faculty of Medicine, Lucian Blaga University of Sibiu, Sibiu, Romania; 3 Department of Trauma, Hand and Reconstructive Surgery and Orthopedics, University Hospital Jena, Jena, Germany; 4 Department of Traumatology and Orthopaedics, Bürgerspital Solothurn, Solothurn, Switzerland; 5 Bulgarian Academy of Sciences, Bulgarian Academy of Sciences, Institute of Metal Science “Acad. A. Balevski”, Sofia, Bulgaria

**Keywords:** Chauffeur, Radius, Screw, K-wire, Fracture

## Abstract

**Purpose:**

Chauffeur fractures represent a distinctive subset of distal radius fractures and typically arise from a high-energy axial load or a direct blow to the volar aspect of the wrist. In cases of significant displacement, joint incongruity or functional impairment, surgical intervention becomes crucial. However, up to date there is no consensus about the best osteosynthesis treatment option. Therefore, the aim of this study was to investigate the biomechanical competence of Chauffeur fractures fixed with either one headless cannulated compression screw, two headless cannulated compression screws, or two Kirschner (K-) wires.

**Methods:**

Eighteen right synthetic radii with a simulated Chauffeur fracture were assigned to three groups (*n* = 6) for fixation with either one headless cannulated compression screw (3.0 mm x 36 mm; Group 1), two K-wires (1.8 mm; Group 2), or two headless cannulated compression screws (2.2 mm x 30 mm and 2.2 mm x 36 mm; Group 3). The specimens underwent non-destructive biomechanical testing in neutral position, flexion, and extension, followed by progressively increasing cyclic loading to failure in neutral position with monitoring via motion tracking.

**Results:**

Initial construct stiffness in flexion was significantly lower in Group 3 (202.4 ± 32.8 N/mm) versus both Group 1 (283.6 ± 31.3 N/mm) and Group 2 (275.3 ± 57.8 N/mm), *p* < 0.05. Cycles to failure and failure load in Group 2 (9214 ± 644; 510.7 ± 32.2 N) and Group 3 (8282 ± 973; 464.1 ± 48.7 N) were both significantly higher versus Group 1 (4678 ± 930; 283.9 ± 46.5 N), *p* < 0.05.

**Conclusion:**

Whereas treatment of Chauffeur fractures with one cannulated compression headless screw is not recommended, using two K-wires is a valid alternative to the fixation of such fractures with two headless cannulated compression screws.

## Introduction

Fractures of the distal radius are one of the most common injuries encountered in orthopedic practice [1–6]. They account for 8–16% of all bone injuries [7, 8]. Origins of distal radius injuries are multifactorial and can comprise falls, sports-related injuries, or motor vehicle accidents [9]. Different classification systems have highlighted the evolution of understanding of this type fractures [10, 11]. They typically arise from a high-energy axial load or a direct blow to the volar (palmar) aspect of the wrist. This unique pattern of injury, characterized by a fracture of the radial styloid process and extension into the radial shaft, necessitates tailored treatment strategies [12, 13].

Minimally displaced and stable Chauffeur fractures may be managed non-operatively with immobilization and close follow-up, particularly in low-demand patients with good bone quality [14]. However, significant displacement or functional impairment often necessitates surgical intervention [15]. Surgical management, guided by fracture characteristics and patient factors, includes such options as open reduction and internal fixation (ORIF) or percutaneous pinning, both of which have advanced significantly in recent years [16].

Despite the availability of various techniques for fixation of Chauffeur radial fractures, there is no clear agreement about the best treatment approach. Options such as one-screw fixation, two-screw fixation or K-wire fixation have unique advantages and limitations. The biomechanical stability of Chauffeur fracture fixation significantly varies depending on the osteosynthesis technique used, with two-point fixation methods—using either two cannulated compression headless screws (CCHSs) or two Kirschner (K-) wires—providing superior mechanical performance compared to single-screw fixation.

Therefore, the aim of this study was to analyze the biomechanical competence of three different minimally invasive types of osteosynthesis surrounding this fracture pattern.

## Materials and methods

### Specimens & preparation

**Fig. 1:**
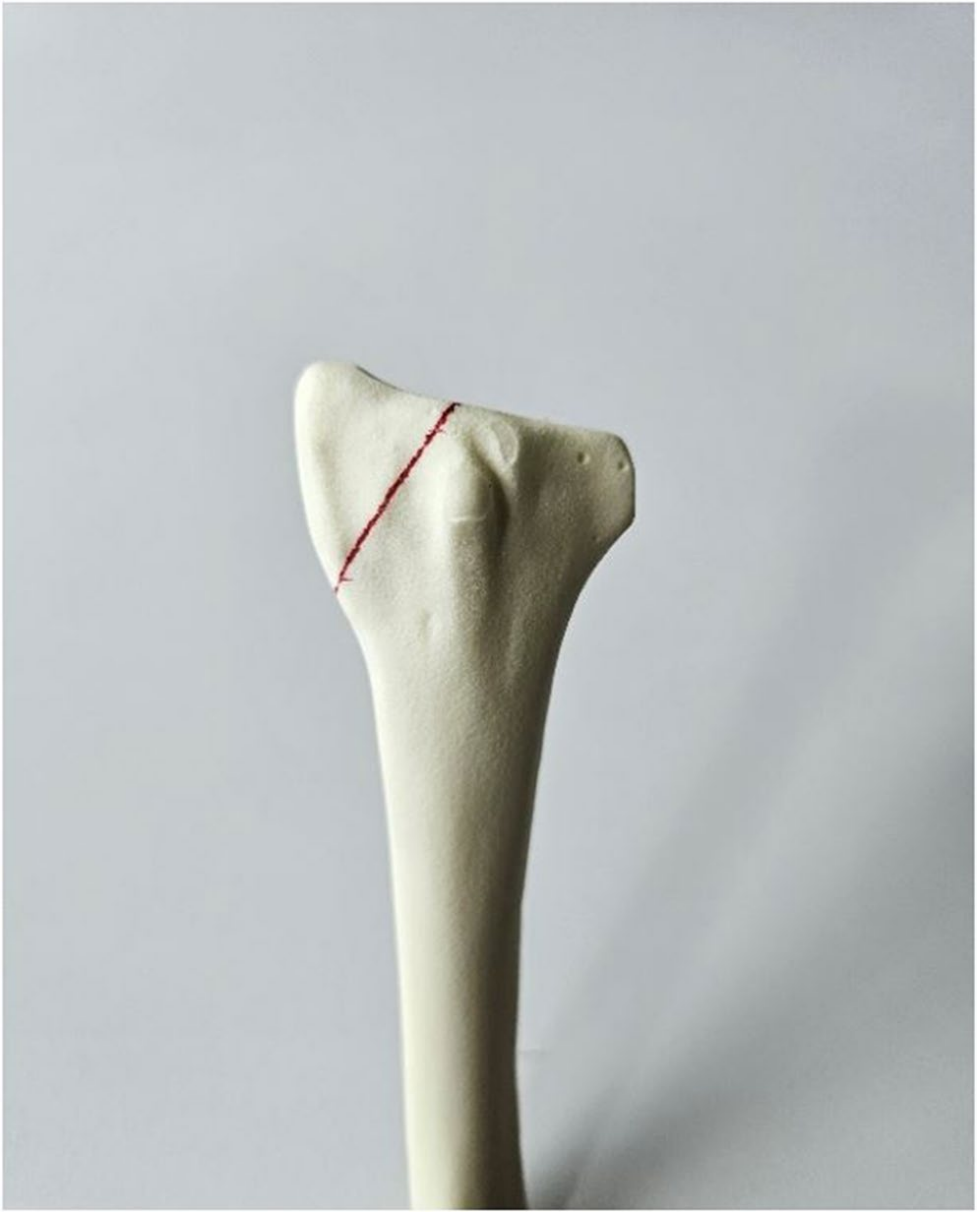
Artificial right radius bone model with marked red line used as guidance to create the osteotomy simulating a Chauffeur fracture

A total of 18 synthetic right radii (#7001, Synbone AG, Zizers, Switzerland) featuring a length of 246 mm and a shaft diameter of 12 mm were considered. A Chauffeur fracture was simulated by means of an oblique osteotomy through the radial styloid process using an oscillating saw. Standardization of the fracture was accounted for by marking the fracture line using equidistant landmarks on the bone surface (Fig. 1).

The osteotomized bone models were then assigned to three groups of six specimens each (*n* = 6) for osteosynthesis with either one 36 mm long 3.0 mm CCHS with a long distal thread in Group 1, two 1.8 mm unthreaded K-wires in Group 2, or two 36 mm and 30 mm long 2.2 mm CCHSs in Group 3, both with a long distal thread. The trajectory of the single screw in Group 1, one of the two K-wires in group 2, and the 36 mm long screw in Group 3, was consistent across the groups, featuring an entry point at the apex of the styloid process, and processing further in coronal plane perpendicularly to the fracture plane. Furthermore, the trajectory of the other K-wire in Group 2 and the 30 mm long screw in Group 3 was consistent, starting with the entry point on the lateral aspect of the styloid process, and proceeding parallel to the articular surface, 2 mm below it. Whereas in Group 1 the pilot hole was pre-drilled using a 2.0 mm drill bit, the pilot holes in Group 3 featured a 1.8 mm diameter.

**Fig. 2:**
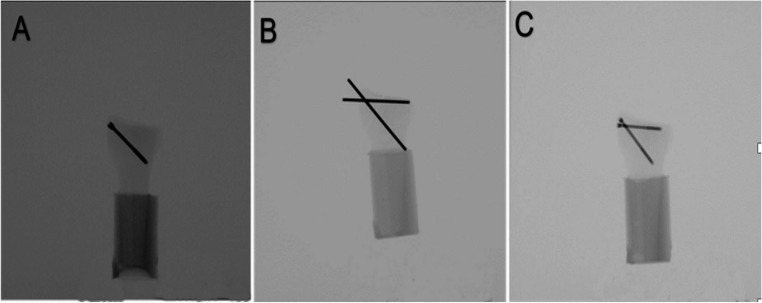
Anteroposterior X-rays post instrumentation visualizing exemplified specimens from Group 1 (**A**), Group 2 (**B**), and Group 3 (**C**)

The instrumentation in all groups was performed by one resident surgeon adhering to the established surgical guidelines, ensuring consistent and accurate placement. Correct implant placement was radiologically confirmed (Fig. 2). All implants were provided by the same manufacturer (Johnson & Johnson MedTech, Zuchwil, Switzerland). Whereas all screws were manufactured from commercially pure titanium, the K-wires were made from cold drawn implant grade stainless steel (316 L). The shaft of each synthetic radius was cut at 90 mm distally from the articular surface and embedded in a cylindrical block made of polymethylmethacrylate (PMMA, SCS-Beracryl D-28, Suter Kunststoffe AG, Fraubrunnen, Switzerland).

### Biomechanical testing

Biomechanical testing was performed on a servo-hydraulic material testing machine (MiniBionix, MTS Systems Corp., Eden Prairie, MN, USA) equipped with a 5 kN load cell (MCS-10-005, HBM, Darmstadt, Germany). The workflow comprised four consecutive biomechanical tests. To simulate different functional movements and stress patterns, in a first phase the specimens were non-destructively tested in simulated neutral position, 40° flexion and 40° extension, followed by the second phase comprising destructive cyclic testing in neutral position.

**Fig. 3:**
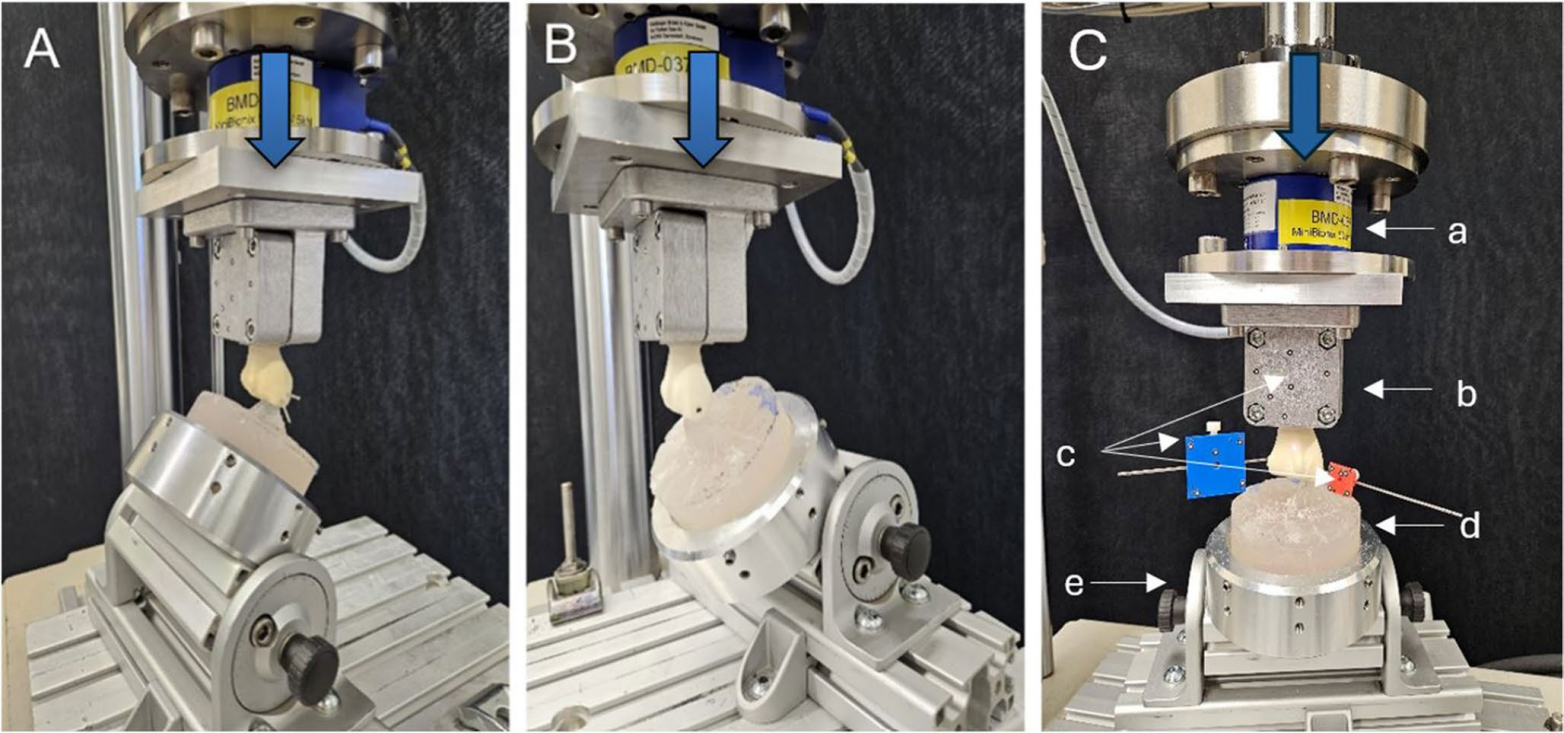
Setup with a specimen mounted for biomechanical testing in extension (40°) (**A**), flexion (40°) [19] (**B**) and neutral position (**C**). Vertical arrow denotes loading direction. (**C**) (**a**) load cell, (**b**) shaft fixation, (**c**) optical markers sets, (**d**) scaphoid PMMA mold, (**e**) inclinable mold fixation

The radial shaft embedding of each construct was rigidly attached to the machine transducer and the interconnected load cell via a custom made holder. Distally, the Chauffeur fragment was supported on a PMMA mold resembling the complementary scaphoid. The mold rested in a custom tiltable holder attached to the machine base, allowing to adjust the angle of the mold for the corresponding test in neutral position, 40° flexion, or 40° extension (Fig. 3). Optical marker sets were affixed to both the fragment and specimen’s shaft for interfragmentary motion tracking.

For each of the initial three non-destructive tests, three consecutive axial compressive ramps from 5 N to 50 Nat a rate of 4.5 N/s were applied to the constructs.

In the fourth test, destructive cyclic sinusoidal compressive loading was applied at 2 Hz [17, 18], with constant valley load of 10 N and progressively increasing peak load at a rate of 0.05 N/cycle, starting from 50 N. The test stop criterion was set to 15 mm transducer displacement, which was considered sufficient to achieve failure of all specimens.

### Data acquisition & evaluation

Machine data in terms of axial displacement and axial force were continuously acquired during all tests at 100 Hz. Based on the machine data collected during non-destructive testing, construct stiffness was calculated from the load-displacement curves of the last loading ramp of each test within a linear range between 40 N and 50 N.

**Fig. 4:**
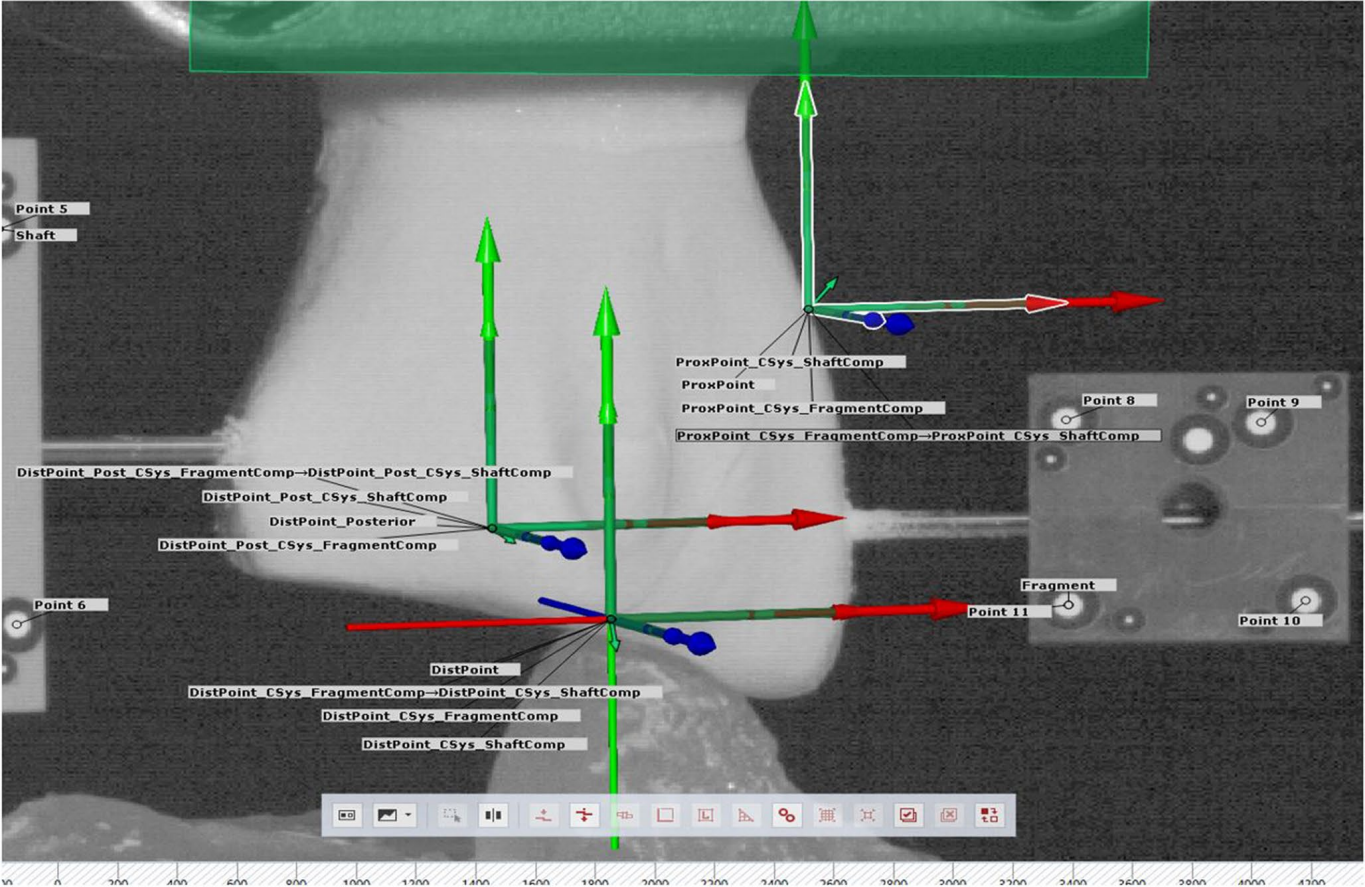
Visualization of the anatomically aligned coordinate system together with the most distal posterior and the most distal anterior fracture aspects of interest created by the GOM software

The coordinates of the attached markers were continuously recorded throughout cyclic testing at 20 Hz using a stereographic camera system (Aramis SRX, Carl Zeiss GOM Metrology GmbH, Braunschweig, Germany) operating at 12 megapixel and a maximum acceptance error of 0.004–0.020 mm [20]. Based on these, total displacement of the most distal posterior and the most distal anterior fracture aspects was calculated as the magnitude of their movements along the three principal axes of an anatomically aligned coordinate system (Fig. 4). In addition, fragment rotation around the medio-lateral axis was evaluated.

The total displacement of both fracture aspects and the fragment rotation were evaluated after 500, 1000, 1500, 2000, 2500, 3000, 3500, and 4000 cycles under peak loading conditions relative the values at the beginning of the cyclic test. In addition, a clinically relevant criterion for specimens’ failure was arbitrarily set to 3 mm total displacement of either of the two fracture aspects. Correspondingly, the number of cycles until fulfillment of this criterion—defined as cycles to failure—was calculated together with the respective peak load—defined as failure load.

Statistical analysis was performed with SPSS software package (V.27, IBM, Armonk, NY, USA). Mean value and standard deviation (SD) were calculated to summarize the data’s central tendency and dispersion. The sample size of 6 specimens per group (*n*= 6) was chosen based on a priori power analysis considering a level of significance set at 0.05 and a power of 0.8, assuming that the specimens fixed with 2 CCHSs would fail at 50% higher load on average compared to those fixed with a single CCHS, and that the standard deviation would not exceed 25% of the difference between the mean values. Shapiro-Wilk test was used to screen and prove normality of the data distribution. One-Way Analysis of Variance (ANOVA) and General Linear Model Repeated Measures tests were applied for group comparisons in combination with Bonferroni post-hoc test for multiple comparisons. The level of overall significance was set at 0.05 for all statistical tests.

## Results

**Fig. 5:**
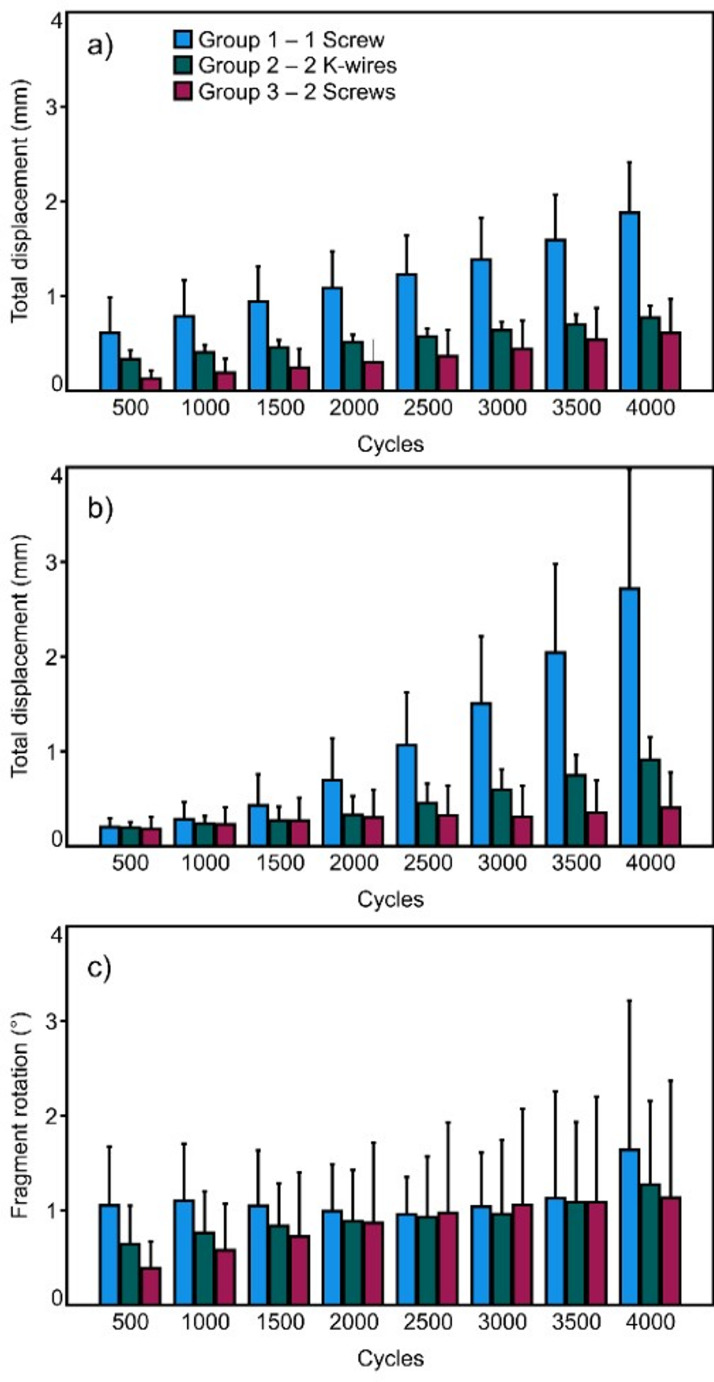
Total displacement of the most distal anterior (**a**) and most distal posterior (**b**) fracture aspects, and fragment rotation (**c**) over the first 4000 test cycles, presented for each group separately in terms of mean value and standard deviation

Construct stiffness in neutral position was highest in Group 3 (277.8 ± 84.6 N/mm) (mean ± SD), followed by Group 1 (249.0 ± 101.0 N/mm) and Group 2 (216.4 ± 58.6 N/mm), with no significant differences between the groups, *p* = 0.505. Similarly, construct stiffness in extension was highest in Group 3 (258.2 ± 42.1 N/mm), followed by Group 1 (208.3 ± 54.2 N/mm) and Group 2 (180.6 ± 46.5 N/mm), with no significant differences between the groups, *p* = 0.069. However, construct stiffness in flexion demonstrated highest values in Group 1 (283.6 ± 31.3 N/mm), followed by Group 2 (275.3 ± 57.8 N/mm) and Group 3 (202.4 ± 32.8 N/mm), with significant differences detected between Group 3 and both Group 1 and Group 2, *p* ≤ 0.043, and no significant difference between Group 1 and Group 2, *p* > 0.999. The outcome measures for the total displacement of the most distal anterior and most distal posterior fracture aspects, as well as for the fragment rotation over the first 4000 cycles, are summarized in Fig. 5. The total displacement of both evaluated fracture aspects was associated with significantly higher values in Group 1 versus both Group 2 and Group 3 (*p* ≤ 0.029 ), with no significant difference between Group 2 and Group 3 (*p* ≥ 0.850). The progress over the first 4000 cycles was associated with a significant increase for each group and parameter (*p* ≤ 0.006), except for the posterior fracture aspect in Group 3 (*p* = 0.162). On the other hand, fragment rotation remained non-significantly different between the groups (*p* = 0.752) and demonstrated no significant changes over cycles within each group (*p* ≥ 0.101).

Cycles to failure and failure load were highest for Group 3 (9214 ± 644 cycles/510.7 ± 32.2 N), followed by Group 2 (8282 ± 973 cycles/464.1 ± 48.7 N) and Group 1 (4678 ± 930 cycles/283.9 ± 46.5 N), with significantly higher values in both Group 2 and Group 3 versus Group 1 (*p* ≤ 0.001), and with no significant difference between Group 2 and Group 3, *p* = 0.278.

**Fig. 6:**
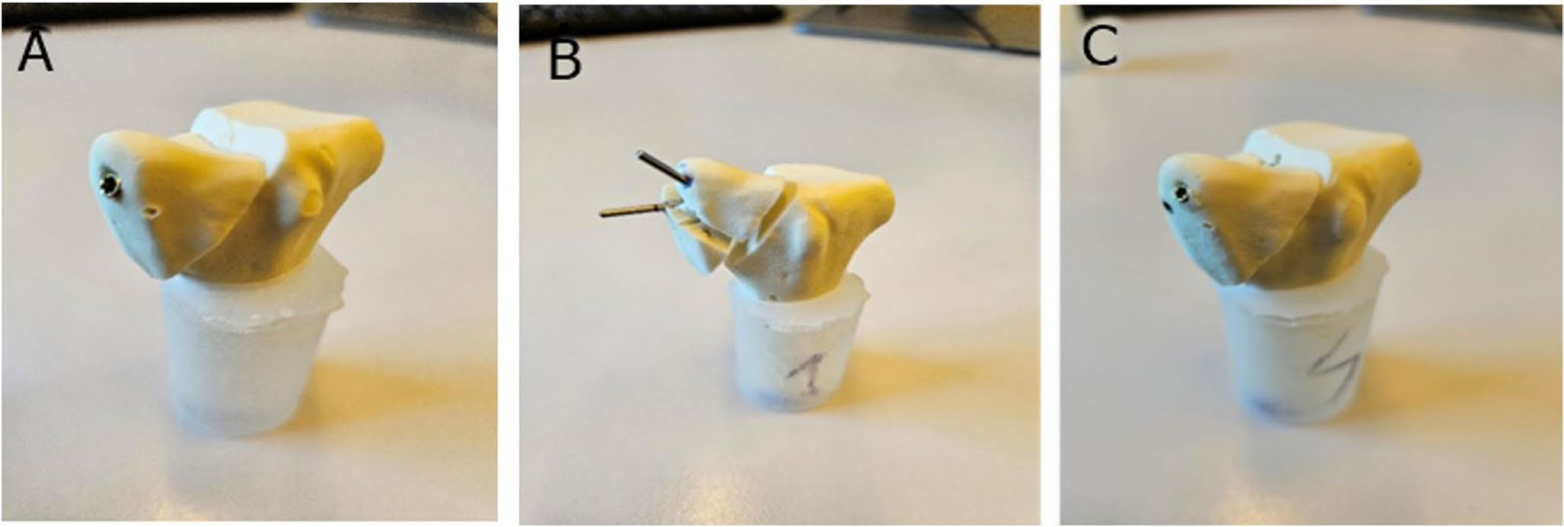
Post-test images of specimens from Group 1 (**A**), Group 2 (**B**), and Group 3 (**C**)

**Fig. 7:**
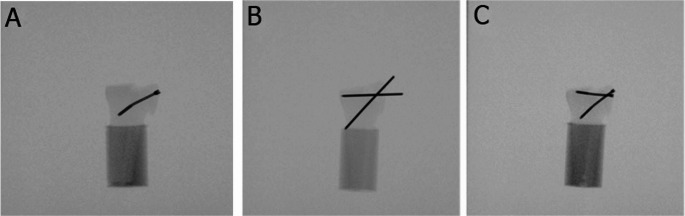
Post-test X-Rays of specimens from Group 1 (**A**), Group 2 (**B**), and Group 3 (**C**)

The failure types were characterized by a radius styloid fracture and implant cut-out in Group 2, and implant bending in Group 1 and Group 3 (Figs. 6 and 7).

## Discussion

Initial construct stiffness in flexion was significantly lower in Group 3 compared to both Group 1 and Group 2. Despite this, both Group 3 and Group 2 demonstrated significantly higher cycles to failure and failure loads versus Group 1. This indicates that single-screw fixation offers the least biomechanical stability. Numerous studies have contributed to valuable insights into the management of distal radius fractures, including Chauffeur fractures. Meena et al. [21] conducted a retrospective analysis of Chauffeur fractures, highlighting the importance of accurate diagnosis, appropriate classification, and tailored treatment plans. Additionally, Hassellund et al. [22] and Teimouri et al. [23] compared the outcomes of surgical versus non-operative approaches in distal radius fractures, emphasizing the significance of individualized decision-making based on fracture characteristics and patient factors.

The observed lower initial construct stiffness associated with two K-wires in the simulated flexion test might have been attributed to the mechanical properties of the K-wires themselves, which could allow for more deformation under these loading conditions. While this could be perceived as a limitation, it is important to note that moderate flexibility could have its merits in certain clinical scenarios, potentially enabling better load distribution and reducing stress concentration at the fracture site.

The significant increase in fragment displacement observed in Group 1 over 4000 cycles, as compared to both Group 2 and Group 3, raises concerns about the construct’s stability and durability under cyclic loading. This finding suggests that the one-screw configuration might be more prone to deformation and fatigue over time, potentially compromising its integrity and clinical functionality. Moreover, the notably lower cycles to failure associated with the one-screw group indicate its limited ability to withstand repeated loading before reaching a critical displacement threshold.

Despite the lower initial stiffness observed with two K-wires in simulated flexion, it is oteworthy that these constructs exhibited comparable performance to two screws under cyclic loading. This finding implies that while the construct may have exhibited greater flexibility initially, it withstood the cyclic loading conditions equally well in terms of displacement and durability. This indicates the robustness of the two K-wires configuration in maintaining its stability and integrity over repeated loading cycles. Sanders et al. affirmed in their study that three K-wires are better for percutaneous pinning, however, they also reported that two K-wires are also very stable [24].

The comparable performance between two K-wires and two screws can raise questions about the influence of material composition on outcomes. Stainless steel and titanium have distinct mechanical and biological properties that could impact fixation stability. Stainless steel is generally more rigid, providing higher strength and stability, which might prevent micro-movements at the fracture site. In contrast, titanium, with its superior biocompatibility and lower modulus of elasticity, reduces stress shielding, potentially compensating for its lesser rigidity.

The study conducted by Meng et al. on a cohort of 48 patients reported that percutaneous fixation with cemented K-wire frame is a safe and preferred choice for treatment of Chauffeaur fractures [14].

In the study conducted by Thanapong Waitayawinyu et al., fragment-specific plates and three -headless screws fixations provided superior stiffness [25].

Based on the collective findings, the use of 2 K-wires emerges as a viable alternative to the 2- screw configuration, particularly in scenarios where a degree of flexibility may be advantageous. On the other hand, the less favorable performance of the 1-screw configuration under cyclic loading suggests that its utilization should be reconsidered. The observed equivalent performance of 2 K-wires and 2 screws under cyclic loading reinforces the notion that both options could be suitable for promoting construct stability while accounting for clinical preferences.

In the context of Chauffeur fracture osteosynthesis, it is well-established that the use of locking plates with screws demonstrates superior efficacy compared to osteosynthesis with headless compression screws (HCSs) or K-wires, although the latter two methods offer specific advantages. Osteosynthesis utilizing HCS or K-wires is associated with a reduced operative duration relative to alternative osteosynthesis techniques, a critical benefit for polytraumatized and hemodynamically unstable patients, while still achieving favorable clinical outcomes. The percutaneous approach which obviates the need for a large incision effectively lowers the risk of infection and wound dehiscence. Furthermore, the deployment of CCHSs presents a lower risk of interference with surrounding soft tissues, such as tendons or muscles. The surgical instrumentation required is streamlined and the associated learning curve is minimal.

The lack of significant differences between groups regarding fragment rotation indicates that the choice of instrumentation did not have a pronounced impact on the angular deformations experienced by the fragment during cyclic loading. This suggests that the biomechanical behavior of the constructs was not significantly influenced by their instrumentation in terms of fragment rotation. The use of two-point fixation with 2 K-wires or 2 screws did not impact rotational stability when compared to a single screw. This outcome might have been influenced by the specific test setup. Other studies suggest that the use of locking plates for treatment of distal radius fractures provide improved functional outcomes [26–29], while others say that there is no difference between K-wires fixation and locking plate fixation [26].

Another important point in this study is the usage of CCHS. Headless screws can be countersunk below the bone surface, reducing the risk of soft tissue irritation and potential complications arising from hardware prominence. This minimizes the chances of discomfort, swelling, and limited joint mobility often associated with traditional screw designs. The headless design allows for better contouring to the bone’s anatomy, facilitating accurate reduction and alignment of the fracture fragments. This anatomical adaptation ensures optimal contact between bone and implant, promoting stability and facilitating natural joint movement. Moreover, screw fixation usually involves smaller incisions and less stripping of periosteum, which helps preserve the local blood supply to the bone. This can promote better bone healing and reduce the risk of such complications as avascular necrosis.

The limitations of this study were that all instrumentations and measurements were conducted in a laboratory on synthetic bones. A synthetic bone model does not fully replicate the physiological behavior of a real bone due to several limitations such as lack of biological content, difference in mechanical characteristics, and material discrepancies or absence of soft tissue interaction.

To enhance the comprehensiveness of the study, future research could consider study design with use of human cadaveric bones. This approach would provide more accurate and applicable data. Other limitations include missing information on how much stability is enough for healing.

Important advantages of this study include the application of precise motion tracking enabling highly accurate assessment of micro-movements at the fracture site and the implementation of cyclic loading to reflect better the physiological conditions rather than applying quasi-static loading.

## Conclusions

Due to its inferior biomechanical performance, the use of a single cannulated compression headless screw for treatment of Chauffeur fractures is not recommended. On the other hand, using two K-wires is a valid alternative to the fixation of such fractures with two cannulated compression headless screws.

## Data Availability

All data relevant to the study are included in the article.

## Abbreviations

HCS: Headless compression screw
K-: Kirschner
ORIF: Open reduction and internal fixation
CCHS: Cannulated Compression Headless Screw
PMMA: polymethylmethacrylate
SD: standard deviation
ANOVA: One-Way Analysis of Variance
GLM: General Linear Model
RM: Repeated Measures
